# Inflammation in osteonecrosis of the femoral head: Evaluation of hematological biomarkers and potential prognostic value

**DOI:** 10.1371/journal.pone.0346158

**Published:** 2026-03-27

**Authors:** Tran Canh Tung Nguyen, Dao Doan Trinh Phan, Thi Huyen Do, Thanh Nam Luong, Hoang Khang Le

**Affiliations:** 1 Department of Trauma and Orthopaedic Surgery, Military Hospital 103, Vietnam Military Medical University, Hanoi, Vietnam; 2 Faculty of Medicine, Vietnam Military Medical University, Hanoi, Vietnam; University of Montenegro-Faculty of Medicine, MONTENEGRO

## Abstract

Increasing evidence suggests that inflammation and immune imbalance are associated with osteonecrosis of the femoral head (ONFH). This study aimed to investigate peripheral inflammatory biomarkers in patients with nontraumatic ONFH and assess their potential prognostic value. A retrospective analysis was conducted on 80 patients with nontraumatic ONFH and 110 control individuals without ONFH. Peripheral blood cell counts, C-reactive protein (CRP), and derived inflammatory indices including neutrophil-to-lymphocyte ratio (NLR), monocyte-to-lymphocyte ratio (MLR), platelet-to-lymphocyte ratio (PLR), and systemic immune-inflammation index (SII) were compared between groups and among different disease stages. Diagnostic performance was assessed using receiver operating characteristic (ROC) curve analysis. The results showed that ONFH patients showed significantly higher CRP, monocyte count (cM), monocyte percentage (pM), MLR, and platelet count (PLT). ROC analysis revealed that CRP (AUC = 0.700, sensitivity = 71.3%, specificity = 65.5%) and cM (AUC = 0.690, sensitivity = 82.5%) had the highest diagnostic accuracy. CRP levels differed significantly across disease stages, peaking at stage II (P = 0.0107), while other indices remained stable. These findings support the concept that both systemic and local inflammatory mechanisms contribute to the pathogenesis of ONFH. CRP and monocyte indices may serve as inexpensive, accessible adjuncts for early disease screening and monitoring.

## Introduction

Osteonecrosis of the femoral head (ONFH), also known as avascular necrosis, is characterized by bone cell death resulting from disrupted blood flow to the subchondral bone from a traumatic or nontraumatic origin, leading to necrosis, structural collapse of the femoral head, hip joint deformity, and consequent loss of mobility. This disease is thought to occur more frequently in Asian populations with approximately 0.0182% in Japan and 0.0289% in South Korea, predominantly affecting adults between 30 and 60 years of age [1–4]. ONFH progresses from early to advanced stages without regression, and once structural collapse occurs, conservative treatment becomes ineffective [3]. Consequently, this condition imposes a substantial burden on patients’ quality of life and contributes significantly to socioeconomic and healthcare challenges.

The pathogenesis of ONFH remains incompletely understood. Several inflammatory cytokines have been linked to this disease, including interleukin-34 (IL-34), which promotes osteoclast differentiation via ERK, STAT3, and non-canonical NF-κB signaling, and IL-4, whose gene polymorphisms have been associated with increased susceptibility to steroid-induced ONFH [5–7]. Notably, Mendelian randomization analysis has provided genetic evidence linking immune dysregulation to osteonecrosis, identifying monocyte-related immune factors as causally associated with disease susceptibility [8]. In particular, pro-inflammatory monocyte subsets, characterized by increased secretion of tumor necrosis factor alpha (TNF-α) and IL-1β, may contribute to bone tissue damage and osteonecrotic progression [9,10]. Taken together, these findings raise the possibility that inflammatory and immune imbalance might be involved in the pathogenesis of ONFH. Thus, evaluating accessible systemic inflammatory markers may provide insights into systemic inflammatory responses associated with ONFH.

Peripheral blood testing is a simple, cost-effective, and widely available diagnostic tool that provides valuable information about systemic inflammatory status. Conventional hematologic parameters such as white blood cell (WBC) count, leukocyte differentials, and C-reactive protein (CRP) are routinely used to assess the presence and severity of inflammation in clinical practice. In recent years, several hematologic ratios derived from routine blood counts have attracted attention as indicators reflecting the imbalance between inflammation and immune regulation, including the neutrophil-to-lymphocyte ratio (NLR), monocyte-to-lymphocyte ratio (MLR), and platelet-to-lymphocyte ratio (PLR) [11–16]. These indices are inexpensive, widely available, and have been shown to correlate with disease activity and prognosis in various inflammatory and degenerative disorders, including rheumatoid arthritis, osteoarthritis, and intervertebral disc degeneration [11–13,16,17]. However, the diagnostic and stage-specific value of these hematologic biomarkers in nontraumatic ONFH remains insufficiently characterized. The objective of this study is to investigate the possible diagnostic value of these parameters in diagnosis and staging in patients with ONFH, thereby facilitating early clinical diagnosis and prevention strategies.

## Materials and methods

### Study design and participants

The retrospective study was carried out at the Joint Surgery Department of the Trauma and Orthopaedics Center, Military Hospital 103, Vietnam Military Medical University between the 1^st^ of January 2023 and the 1^st^ of October 2025. All procedures were conducted in accordance with the principles of the Declaration of Helsinki. The study was approved by the Institutional Review Board of Military Hospital 103 (approval number 192/HDDD, date: June 15^th^, 2023), and the requirement for informed consent was waived due to its retrospective design. All consecutive patients who were diagnosed with ONFH were enrolled. The diagnosis of ONFH was established based on clinical presentation and imaging findings, and disease stages were classified according to the Ficat and Arlet classification [18]. All patients with traumatic ONFH, a history of previous hip surgery, incomplete or inadequate diagnostic or treatment records, or missing or incomplete clinical or laboratory data were excluded. The control group consisted of individuals who presented to the hospital for bone mineral density assessment during the same period. These subjects had no clinical or radiological evidence of femoral head necrosis and had completed all routine hematologic and biochemical examinations. Any ONFH patient or control subject with immune system diseases such as rheumatoid arthritis (RA) or ankylosing spondylitis (AS), concomitant systemic or local infections, severe systemic disorders affecting vital organs, severe liver disease, or malignant tumors was also excluded.

### Data collection

Data were retrospectively extracted from the electronic medical record system for research purposes from the 14^th^ of October 2025. At inclusion, data were deidentified before handing to the data collector. Demographic variables, including age, sex, height, weight, body mass index (BMI), as well as comorbidities including hypertension, diabetes mellitus, and other chronic diseases, were recorded to evaluate potential confounding effects.

Peripheral blood biomarkers were also collected for analysis. Peripheral blood routine examinations were automatically performed using AU5800 or AU680 analyzers (Beckman Coulter, Indianapolis, IN, USA). Parameters, including WBC count, neutrophil count and percentage (cN and pN), lymphocyte count and percentage (cL and pL), monocyte count and percentage (cM and pM), eosinophil count and percentage (cE and pE), basophil count and percentage (cB and pB), platelets (PLT), and serum CRP levels, were obtained from the laboratory database. Inflammatory indices were calculated as follows: NLR = cN/ cL, MLR = cM/ cL, PLR = PLT/ cL, and systemic immune inflammation index (SII) = PLT × cN/ cL.

### Statistical analysis

All statistical analyses were performed using GraphPad Prism version 10.0 (GraphPad Software, San Diego, USA). Categorical variables (sex, comorbidities) were expressed as frequencies and percentages and compared using the chi-square test. Continuous variables (age, BMI, and hematologic parameters) were expressed as mean ± standard error of the mean (SEM). Comparisons between the ONFH and control groups were analyzed using the independent-samples Student’s t-test, while comparisons among the four ONFH stages were performed using one-way analysis of variance (ANOVA). Diagnostic performance of individual biomarkers was evaluated using receiver operating characteristic (ROC) curve analysis, and the area under the curve (AUC) was calculated. Optimal cutoff values were determined using the maximum Youden index (J = sensitivity + specificity − 1), as no established clinical thresholds currently exist for these biomarkers in ONFH. Sensitivity, specificity, and corresponding 95% confidence intervals were calculated for each parameter. A P value < 0.05 was considered statistically significant.

## Results

### Baseline characteristics of patients with ONFH and the controls

A total of 80 patients with nontraumatic ONFH (64 males and 16 females, mean age 59.60 ± 1.65 years) and 110 controls (84 males and 26 females, mean age 59.93 ± 1.53 years) participated in this study. Baseline characteristics of the participants are summarized in Table 1. There were no significant differences in demographic information, including age, sex, height, weight, BMI, and medical comorbidities between the two groups.

**Table 1:** Comparison of baseline characteristics between the ONFH group and the control group.

	ONFH	Control	P value
Number of patients, n	80	110	
Sex, n (%)			0.55
Male	64 (80)	84 (76.36)	
Female	16 (20)	26 (23.64)	
Age (year)	59.60 ± 1.65	59.93 ± 1.53	0.89
Height (m)	1.65 ± 0.01	1.64 ± 0.01	0.33
Weight (kg)	61.35 ± 1.04	60.91 ± 0.92	0.75
BMI (kg/m²)	18.58 ± 0.27	18.62 ± 0.25	0.91
Medical comorbidities, n (%)			
Hypertension	21 (26.25)	33 (30)	0.57
Diabetes	7 (8.75)	18 (16.36)	0.13
Other diseases	33 (41.25)	48 (43.64)	0.74

### Comparison of peripheral blood biomarkers between the two groups

The preoperative peripheral blood parameters, including WBC, pN, cN, pE, cE, pL, cL, pB, cB, pM, cM, PLT, PLR, NLR, MLR, SII, and CRP, were compared between the ONFH and control groups. As shown in Table 2, patients with ONFH exhibited significantly higher pM (P < 0.0001), cM (P < 0.0001), and CRP levels (P < 0.0001) compared with the control group. PLT (P = 0.04) and MLR (P = 0.0012) were also significantly higher in the ONFH group. In contrast, there were no significant differences between the two groups in total WBC count, neutrophil, lymphocyte, eosinophil, basophil parameters, NLR, PLR, or SII (P > 0.05).

**Table 2:** Comparison of peripheral blood biomarkers between the ONFH group and the control group.

	ONFH	Control	P value
WBC (x10^9^/L)	7.80 ± 0.22	7.44 ± 0.15	0.16
pL (%)	28.87 ± 1.05	30.21 ± 0.84	0.31
cL (x10^9^/L)	2.20 ± 0.08	2.17 ± 0.06	0.76
pN (%)	58.62 ± 1.39	59.64 ± 0.93	0.54
cN (x10^9^/L)	4.75 ± 0.18	4.50 ± 0.14	0.27
pM (%)	8.23 ± 0.22	6.93 ± 0.14	<0.0001
cM (x10^9^/L)	0.63 ± 0.02	0.51 ± 0.01	<0.0001
pE (%)	2.67 ± 0.28	3.07 ± 0.26	0.29
cE (x10^9^/L)	0.20 ± 0.02	0.22 ± 0.02	0.64
pB (%)	0.49 ± 0.03	0.48 ± 0.02	0.65
cB (x10^9^/L)	0.031 ± 0.004	0.026 ± 0.003	0.25
PLT (x10^9^/L)	267.10 ± 8.55	246.50 ± 5.48	0.04
NLR	2.46 ± 0.17	2.33 ± 0.13	0.55
MLR	0.31 ± 0.02	0.25 ± 0.01	0.0012
PLR	132.60 ± 6.65	122.20 ± 4.12	0.19
SII	652.60 ± 57.01	567.40 ± 31.17	0.19
CRP (mg/L)	3.79 ± 0.38	1.81 ± 0.18	<0.0001

WBC: white blood cell; pL: percentage of lymphocytes; cL: count of lymphocytes; pN: percentage of neutrophils; cN: count of neutrophils; pM: percentage of monocytes; cM: count of monocytes; pE: percentage of eosinophils; cE: count of eosinophils; pB: percentage of basophils; cB: count of basophils; PLT: platelet; NLR: ratio of neutrophil to lymphocyte count; MLR: ratio of monocyte to lymphocyte count; PLR: ratio of platelet to lymphocyte count; SII: systemic immune inflammation index; CRP: C-reactive protein.

### ROC curves analysis of CRP, cM, pM, MLR, and PLT for predicting ONFH

**Fig 1:**
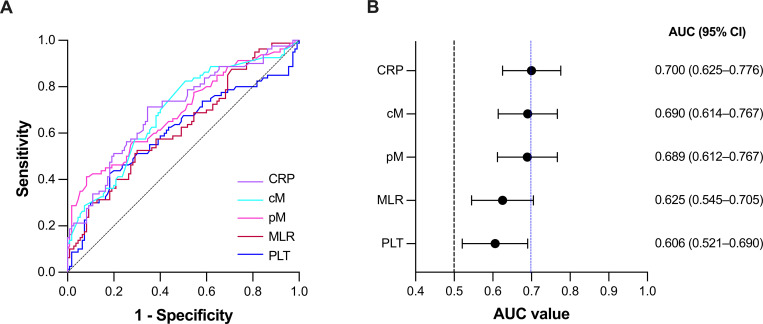
Diagnostic performance of CRP, cM, pM, MLR, and PLT for predicting ONFH. **(A)** ROC curve analysis for predicting ONFH. **(B)** Forest plot summarizing AUC for each biomarker. Chance levels are indicated by a dotted line (AUC = 0.5), and the AUC of the best-performing biomarker is indicated by a dashed blue line. Error bars represent 95% CI. ROC: receiver operating characteristic; AUC: area under the curve; CI: confidence interval; CRP: C-reactive protein; cM: count of monocytes; pM: percentage of monocytes; MLR: ratio of monocyte to lymphocyte count; PLT: platelet.

To further assess the diagnostic performance of these biomarkers, ROC curve analyses were conducted (Table 3 and Fig 1). Among the tested parameters, CRP demonstrated the highest diagnostic performance (AUC = 0.700, P < 0.0001), followed by cM (AUC = 0.690, P < 0.0001), pM (AUC = 0.689, P < 0.0001), MLR (AUC = 0.625, P = 0.003), and PLT (AUC = 0.606, P = 0.013). The optimal cutoff value for CRP was 1.51 mg/L, yielding a sensitivity of 71.25% and specificity of 65.45%. For monocyte count and percentage, the best cutoff values were 0.50 G/L and 8.75%, respectively, with corresponding sensitivities of 82.50% and 41.25%, and specificities of 49.09% and 91.82%. The optimal MLR cutoff was 0.28 (sensitivity = 52.50%, specificity = 70.00%), while the PLT cutoff was 286.50 G/L (sensitivity = 42.50%, specificity = 81.82%).

**Table 3:** ROC curves analysis of CRP, cM, pM, MLR, and PLT for predicting ONFH.

	AUC	95% CI	Youden index	Cutoff value	Sensitivity (%)	Specificity (%)	P value
CRP	0.700	0.625–0.776	0.367	1.51 mg/L	71.25	65.45	<0.0001
cM	0.690	0.614–0.767	0.3159	0.50 x 10^9^/L	82.50	49.09	<0.0001
pM	0.689	0.612–0.767	0.3307	8.75%	41.25	91.82	<0.0001
MLR	0.625	0.545–0.705	0.225	0.28	52.50	70.00	0.003
PLT	0.606	0.521–0.690	0.2432	286.50 x10^9^/L	42.50	81.82	0.013

ROC: receiver operating characteristic; AUC: area under the curve; CI: confidence interval; CRP: C-reactive protein; cM: count of monocytes; pM: percentage of monocytes; MLR: ratio of monocyte to lymphocyte count; PLT: platelet.

### Comparison of peripheral blood biomarkers between the ONFH subgroups

The peripheral blood biomarkers were compared among 80 patients in four ONFH subgroups according to the Ficat and Arlet classification (Table 4). There were no statistically significant differences between the four stages in terms of WBC, pN, cN, pE, cE, pL, cL, pB, cB, pM, cM, PLT, PLR, NLR, MLR, SII. A significant difference was observed in CRP levels among the stages (P = 0.0107), with mean values of 2.37 ± 0.55 mg/L in stage I, 5.71 ± 1.24 mg/L in stage II, 4.61 ± 0.63 mg/L in stage III, and 2.75 ± 0.65 mg/L in stage IV.

**Table 4:** Comparison of peripheral blood biomarkers between the ONFH subgroups.

	Stage I	Stage II	Stage III	Stage IV	P value
WBC (x10^9^/L)	7.94 ± 0.48	8.32 ± 0.59	7.29 ± 0.35	8.17 ± 0.39	0.31
pL (%)	30.65 ± 1.83	28.19 ± 1.88	29.37 ± 2.03	26.49 ± 2.20	0.58
cL (x10^9^/L)	2.36 ± 0.15	2.30 ± 0.18	2.12 ± 0.13	2.11 ± 0.18	0.60
pN (%)	56.34 ± 2.48	59.58 ± 2.47	56.86 ± 2.82	63.43 ± 2.32	0.26
cN (x10^9^/L)	4.67 ± 0.40	4.99 ± 0.44	4.42 ± 0.27	5.22 ± 0.35	0.37
pM (%)	8.09 ± 0.30	8.91 ± 0.63	8.34 ± 0.44	7.74 ± 0.43	0.46
cM (x10^9^/L)	0.64 ± 0.04	0.72 ± 0.06	0.58 ± 0.04	0.63 ± 0.04	0.19
pE (%)	3.11 ± 0.69	2.83 ± 0.72	2.78 ± 0.46	1.90 ± 0.35	0.50
cE (x10^9^/L)	0.24 ± 0.05	0.24 ± 0.07	0.20 ± 0.04	0.16 ± 0.03	0.64
pB (%)	0.47 ± 0.06	0.49 ± 0.07	0.56 ± 0.05	0.44 ± 0.04	0.42
cB (x10^9^/L)	0.03 ± 0.01	0.03 ± 0.01	0.04 ± 0.01	0.02 ± 0.01	0.79
PLT (x10^9^/L)	276.60 ± 16.58	248.10 ± 14.60	261.70 ± 15.02	278.20 ± 20.12	0.67
NLR	2.22 ± 0.36	2.29 ± 0.25	2.37 ± 0.23	2.97 ± 0.48	0.43
MLR	0.30 ± 0.04	0.33 ± 0.03	0.30 ± 0.03	0.35 ± 0.05	0.77
PLR	127.20 ± 13.18	117.80 ± 14.00	131.60 ± 8.65	150.10 ± 19.31	0.49
SII	637.50 ± 126.80	570.70 ± 79.73	579.50 ± 43.23	845.90 ± 190.00	0.32
CRP (mg/L)	2.37 ± 0.55	5.71 ± 1.24	4.61 ± 0.63	2.75 ± 0.65	0.01

WBC: white blood cell**;** pL: percentage of lymphocytes; cL: count of lymphocytes; pN: percentage of neutrophils; cN: count of neutrophils; pM: percentage of monocytes; cM: count of monocytes; pE: percentage of eosinophils; cE: count of eosinophils; pB: percentage of basophils; cB: count of basophils; PLT: platelet; NLR: ratio of neutrophil to lymphocyte count; MLR: ratio of monocyte to lymphocyte count; PLR: ratio of platelet to lymphocyte count; SII: systemic immune inflammation index; CRP: C-reactive protein.

## Discussion

In this retrospective study, we compared the peripheral blood biomarkers between ONFH patients and control individuals to elucidate the underlying inflammatory mechanisms as well as explore their potential diagnostic value. The results showed that patients with ONFH exhibited significantly elevated levels of CRP, cM, pM, MLR, and PLT compared with non-ONFH controls. ROC curve analysis revealed that CRP and cM demonstrated the greatest diagnostic accuracy among these markers. Moreover, CRP levels varied significantly among different Ficat and Arlet stages, peaking in stage II, whereas other hematologic indices remained relatively stable. Collectively, these findings suggest that inflammation and immune dysregulation might play an important role in the pathophysiology and clinical progression of ONFH.

CRP is one of the most widely used acute-phase biomarkers for detecting systemic inflammation and tissue injury. In our study, although the increase in CRP of the patients with ONFH was modest, it was significantly different compared to individuals without ONFH. To our knowledge, no studies have evaluated CRP levels in ONFH in comparison with healthy controls; however, previous reports have demonstrated increased CRP levels in ONFH patients. In patients with Takayasu arteritis, higher CRP levels were associated with the presence of ONFH, suggesting that inflammation may contribute to small vessel involvement supplying the femoral head and thereby trigger femoral head necrosis [19]. Similarly, in other high-risk settings such as HIV associated ONFH, CRP has also been reported to be higher at diagnosis compared with controls [20]. In addition, a recent study evaluating inflammatory markers before primary total hip arthroplasty found that, although CRP values remained within the normal range, a preoperative diagnosis of ONFH was independently associated with a higher likelihood of CRP elevation compared with hip arthritis [21]. Taken together, these findings are consistent with our observation and suggest the presence of a low-grade systemic inflammatory response in patients with ONFH.

Pro-inflammatory cytokines, such as IL-6, IL-1β, and TNF-α are responsible for the induction of CRP synthesis in the liver [22]. Recent studies have identified additional cytokines, such as IL-34 and IL-4, as key immunomodulators in ONFH [6,7]. IL-34, a ligand for the colony-stimulating factor-1 receptor CSF-1R, promotes monocyte survival and differentiation into osteoclasts via ERK and NF-κB signaling pathways, thereby exacerbating bone resorption and necrotic collapse [6,23]. Conversely, IL-4 exerts an anti-inflammatory influence by driving macrophage polarization toward the reparative M2 phenotype and suppressing the transcription of IL-1β and TNF-α [7,24]. Genetic variants of IL-4 have been associated with impaired anti-inflammatory signaling, predisposing individuals to steroid-induced ONFH [7]. Thus, these cytokine imbalances may sustain chronic low-grade inflammation, endothelial injury, and osteoclastic overactivity, all of which contribute to necrotic degeneration of the femoral head. Elevated CRP thus serves as a downstream biomarker of this cytokine-driven immune disturbance.

The interplay between these cytokines also has implications for monocyte activation and macrophage differentiation, as both IL-34 and IL-4 directly regulate the lineage and function of mononuclear phagocytes. Monocytes and macrophages play essential roles in bone homeostasis, immune regulation, and the removal of necrotic debris. They can differentiate into two major functional phenotypes: classically activated M1 macrophages, which produce pro-inflammatory cytokines such as TNF-α, IL-1β, nitric oxide, and reactive oxygen species, and alternatively activated M2 macrophages, which secrete anti-inflammatory cytokines such as IL-10, transforming growth factor-β, and contribute to angiogenesis and tissue remodeling [24,25]. Emerging molecular and immunologic evidence supports the involvement of the monocyte–macrophage axis in disease pathogenesis. A recent research article utilized a Mendelian randomization study to provide genetic evidence for causal associations between a broad spectrum of immune factors and osteonecrosis [8]. It was discovered that the genetically predicted level of a distinct immune factor (CD62L–monocyte%monocyte) showed a notable positive correlation with osteonecrosis, implicating monocyte-driven immune processes in disease development. Similarly, immune infiltration profiling using CIBERSORT identified distinct innate immune signatures in ONFH tissue, including enrichment of macrophage-related pathways that correlate with disease severity [26]. In line with these findings, a bioinformatics study reported that TARDBP expression was significantly elevated in ONFH samples and correlated with monocyte and M1 macrophage infiltration, linking gene-level dysregulation to pro-inflammatory innate immune activity [27]. Although clinical studies evaluating circulating monocyte subsets in ONFH are limited, elevations of monocyte-based inflammatory ratios have been reported in other degenerative musculoskeletal disorders characterized by chronic low-grade inflammation, such as knee osteoarthritis [28], as well as in autoimmune conditions such as axial spondyloarthritis (axSpA) and rheumatoid arthritis (RA) [29,30]. Consistently, our study found elevated circulating cM, pM, and MLR in ONFH patients, indicating a shift toward chronic innate immune activation.

Histopathologic studies have demonstrated that M1 macrophages dominate in early necrotic lesions, where high TNF-α and IL-6 activity drive bone resorption and vascular compromise [31,32]. As the disease progresses, M2 macrophages become more prominent, reflecting attempts at tissue repair and fibrotic remodeling of the necrotic area [31]. Therefore, the elevated MLR observed in our cohort likely represents an imbalance between M1 and M2 activity rather than an acute inflammatory burst. Aligned with this mechanism, when comparing inflammatory markers across disease stages, CRP also showed significant variation, peaking in stage II and declining in later stages. Ficat–Arlet stage II represents the pre-collapse phase, characterized by radiographic sclerosis and cystic changes, while preservation of femoral head contour is maintained [18]. Biologically, this stage is marked by an intensified necrotic–viable interface, bone marrow edema, and active osteoclastic remodeling. In experimental models of ONFH, early lesions are characterized by elevated TNF-α expression and predominance of M1 macrophage infiltration [31]. In human ONFH tissue and plasma, enrichment of IL-6 has also been reported, suggesting that locally generated cytokines may enter the systemic circulation during disease progression [32]. IL-6, a downstream mediator of TNF-α signaling, is a principal inducer of hepatic CRP synthesis. Accordingly, increased local TNF-α and IL-6 activity during the active pre-collapse phase may translate into a measurable systemic response, leading to the highest CRP levels observed at stage II in our cohort. In contrast, stages III and IV are dominated by structural collapse and secondary degenerative changes [18]. Although mechanical deterioration progresses, inflammatory activity may shift toward M2 polarization and fibrotic remodeling, resulting in attenuation of systemic CRP in these stages despite worsening radiographic severity. Taken together, the persistent activation of circulating monocytes and macrophage infiltration in necrotic bone tissue likely sustains chronic low-grade inflammation, while elevated CRP reflects episodes of systemic immune activation corresponding to local tissue destruction. These findings support the concept that ONFH involves both systemic and localized inflammatory processes that evolve dynamically over the disease progression. This dual inflammatory pattern underscores the complex interplay between ischemia, immune dysregulation, and tissue repair in the pathophysiology of ONFH.

From a clinical perspective, early intervention in ONFH typically yields more conspicuous results, as therapeutic strategies aim for joint preservation while postponing total hip arthroplasty [33]. Accordingly, early diagnosis and prompt treatment of osteonecrosis stand paramount [34]. However, diagnosis during the initial stages can be challenging due to the lack of overt clinical symptoms. The observation that CRP peaks during stage II, when structural collapse has not yet occurred, suggests that modest CRP elevation may serve as an adjunctive indicator of an active inflammatory window. During this pre-collapse stage, interventions such as core decompression and biologic augmentation are considered most effective [33]. In addition, although therapeutic strategies targeting TNF-α and macrophage-mediated inflammatory pathways remain investigational, modulation of inflammatory signaling may hold therapeutic potential in this disease stage. Nevertheless, further prospective studies are required to better define the role of CRP in the early detection of ONFH and to validate its stage-dependent variation.

In addition to inflammatory activation, our results also revealed a significant increase in PLT count among patients with ONFH. This elevation may reflect an underlying hypercoagulable state, which has been proposed as a key contributor to the ischemic mechanism of femoral head necrosis. Circulating platelet-derived microparticles have been shown to contribute to microvascular thrombosis in ONFH patients [35], and broader reviews have emphasized that coagulation abnormalities and platelet activation are key risk factors in the development of the disease [36]. Furthermore, activated platelets not only mediate thrombosis but also participate directly in inflammation by releasing vasoactive and pro-inflammatory mediators such as platelet factor 4, RANTES, and IL-8, which can enhance leukocyte recruitment, stimulate monocyte–endothelial interactions, and amplify local cytokine signaling [37,38]. These platelet-mediated pathways may thus contribute to both vascular occlusion and inflammatory amplification within necrotic bone tissue. Although the PLR and SII were not significantly different between groups in our study, the increase in PLT count reinforces the hypothesis that ONFH represents an overlapping state of chronic inflammation and prothrombotic activity, in which impaired perfusion and immune activation synergistically drive femoral head degeneration.

Our diagnostic analysis showed that among routine peripheral blood biomarkers, CRP demonstrated the highest discriminative ability for nontraumatic ONFH (AUC = 0.700, 95% CI 0.625–0.776), followed by cM (AUC = 0.690), pM (AUC = 0.689), MLR (AUC = 0.625), and PLT (AUC = 0.606), respectively. Our results are in line with observations in other inflammatory rheumatic conditions. In ankylosing spondylitis (AS), CRP demonstrated the highest diagnostic performance for identifying the presence of AS, with an AUC of 0.677 [39]. Additionally, Huang et al. examined the diagnostic performance of MLR and PLR in axSpA and reported that MLR achieved an AUC of 0.768 for evaluating axSpA patients, including those with AS [29]. Similarly, in rheumatoid arthritis, MLR demonstrated moderate diagnostic accuracy with an AUC of 0.67, followed by NLR (AUC = 0.61) and PLR (AUC = 0.60) for disease detection [30]. Taken together, although the overall diagnostic accuracy of CRP, cM, pM, MLR, and PLT was moderate, we suggest that these hematologic markers may serve as auxiliary tools in the evaluation of ONFH, given that they are inexpensive and routinely available in clinical practice. Furthermore, the relatively higher sensitivity of CRP (71.25%) and cM (82.50%) suggests that these markers are useful for initial screening, while the higher specificity of pM (91.82%) and PLT (81.82%) may assist in confirming diagnosis or monitoring inflammatory remission. However, their moderate sensitivity and specificity mean they should not replace imaging or core diagnostic criteria but could contribute to risk stratification and screening in clinical practice.

This study has several limitations that should be acknowledged. First, it was conducted as a single-center investigation, which may limit the generalizability of the findings, as patient characteristics and clinical practices may differ across institutions. The retrospective design further limits control over data collection and variable standardization, potentially introducing selection bias and unmeasured confounding despite efforts to adjust for baseline characteristics. Also, the relatively small sample size may have reduced statistical power, particularly for subgroup analyses across disease stages. Second, although patients with traumatic or secondary causes were excluded, potential heterogeneity in underlying etiologies such as corticosteroid use, alcohol intake, or metabolic disorders could not be entirely eliminated. Furthermore, although major inflammatory and autoimmune conditions were excluded, common chronic comorbidities were not used as exclusion criteria and may have influenced inflammatory markers, thereby representing potential residual confounders. However, these conditions are highly prevalent in the ONFH population, and their exclusion might have reduced the representativeness and external validity of the cohort. Baseline comorbidities were therefore systematically recorded and showed no significant differences between the ONFH and control groups. Nevertheless, residual confounding cannot be entirely excluded. Third, only baseline inflammatory parameters were evaluated; therefore, dynamic changes in CRP, monocyte count, and related ratios during disease progression were not assessed. Fourth, we did not include local tissue markers or histopathological correlations, which would have provided more direct evidence linking systemic inflammation to local necrotic activity. Finally, the absence of longitudinal follow-up data precluded evaluation of the predictive value of these biomarkers for disease progression or treatment outcomes. Future prospective multicenter studies with larger cohorts and serial biomarker measurements are warranted to validate and expand upon our findings.

## Conclusion

This study demonstrates that patients with nontraumatic ONFH exhibit significantly elevated levels of CRP, cM, pM, MLR, and PLT compared with individuals without ONFH. These alterations reflect the coexistence of systemic inflammation and chronic immune dysregulation, contributing to the pathogenesis and progression of the disease. Among the examined biomarkers, CRP and monocyte indices showed the best diagnostic performance, suggesting their potential utility as accessible and cost-effective adjunctive tools in the assessment of nontraumatic ONFH. The stage-dependent variation of CRP, with a peak at Ficat stage II, further suggests that these markers may reflect biologically active disease before structural collapse occurs. When interpreted in conjunction with radiologic findings, they may assist in early risk assessment and clinical evaluation. Nevertheless, further prospective multicenter studies involving larger cohorts are necessary to validate their diagnostic and prognostic relevance.

## Supporting information

S1 File Dataset used in this study. (XLSX)
